# MRI-Based Medial Temporal Atrophy May Reflect Amyloid PET Positivity in Cognitively Normal Individuals: A Preliminary Study

**DOI:** 10.7759/cureus.96751

**Published:** 2025-11-13

**Authors:** Heii Arai

**Affiliations:** 1 Department of Psychiatry, ALZCLINIC TOKYO, Tokyo, JPN

**Keywords:** alzheimer’s disease, amyloid pet, biomarkers, cognition, hyppocampus, magnetic resonance imaging, medial temporal lobe

## Abstract

This study investigated whether magnetic resonance imaging (MRI)-based medial temporal morphometry can predict amyloid positron emission tomography (PET) positivity in cognitively normal individuals. Sixteen consecutive participants (eight amyloid PET-positive and eight PET-negative) were retrospectively identified, all within the normal cognitive range (Mini-Mental State Examination 28-30). Amyloid PET was performed using florbetapir, with visual interpretation as the primary diagnostic criterion and Centiloid values as supportive measures. MRI-derived volumetric analysis was conducted using the voxel-based specific regional analysis system for Alzheimer’s disease (AD) to quantify medial temporal atrophy. Although between-group differences in hippocampal indices did not reach statistical significance, the PET-positive group showed a trend toward greater atrophy, and centiloid values correlated positively with medial temporal indices. Receiver operating characteristic analysis indicated fair discrimination, highest for the volume-of-interest-to-gray-matter ratio (area under the curve = 0.83). These preliminary findings suggest that subtle hippocampal alterations on routine structural MRI may mirror early amyloid pathology even before cognitive impairment, supporting the potential of MRI-based morphometric assessment as an adjunctive biomarker for early AD detection.

## Introduction

Alzheimer’s disease (AD) is now conceptualized as a biological continuum in which amyloid β protein (Aβ) accumulation can precede overt cognitive decline by many years. Structural magnetic resonance imaging (MRI) remains a core tool across this continuum, with medial temporal lobe - particularly hippocampal-atrophy established as a robust marker at the dementia stage [1-3]. Quantitative frameworks, such as voxel-based specific regional analysis system for Alzheimer’s disease (VSRAD), enable standardized, patient-level assessment of medial temporal atrophy in clinical practice [4-7].

In parallel, amyloid positron emission tomography (PET) has made it possible to quantify cortical Aβ burden on a common centiloid (CL) scale, facilitating cross-tracer harmonization and thresholding [8]. This integration of imaging modalities raises a clinically relevant question: to what extent do subtle hippocampal changes detectable on routine structural MRI mirror early amyloid pathology in individuals who are cognitively normal? Although several studies have linked elevated amyloid to smaller hippocampal volumes or to faster atrophy in cognitively normal cohorts, the associations are modest and heterogeneous across samples and methods [9-12]. Some reports even suggest that voxel-based morphometry (VBM) confined to medial temporal regions has limited capability to detect Aβ pathology per se, underscoring the need for careful, biomarker-anchored evaluation [13].

Consequently, there remains a practical knowledge gap at the preclinical stage of AD, where individuals show normal cognitive scores (Mental State Examination (MMSE) 28-30) yet may harbor underlying amyloid pathology. To address this, we designed this study with two aims. The primary objective was to determine whether MRI-based medial temporal atrophy indices derived from VSRAD can predict amyloid PET positivity in cognitively normal individuals. The secondary objective was to explore correlations between VSRAD-derived indices and quantitative amyloid PET values expressed in CL units. For conceptual clarity, amyloid PET positivity was defined by visual interpretation, consistent with CL threshold ranges described in the Methods section.

## Materials and methods

Participants

Participants were retrospectively identified from clinical records reviewed in reverse chronological order beginning October 1, 2025. Consecutive individuals meeting the following criteria were included until eight amyloid PET-positive and eight PET-negative cases had been accumulated: (i) cognitively normal range (MMSE): 28-30) and (ii) availability of both amyloid PET and structural MRI acquired within the study timeframe. All participants had no history of neurological or psychiatric disorders.

The study was conducted under institutional review board approval with an opt-out policy for secondary use of de-identified clinical data, in accordance with the Japanese Ethical Guidelines for Medical and Health Research Involving Human Subjects. Written informed consent was obtained from the participants for publication of the details of their medical data and any accompanying images.

Amyloid PET acquisition and evaluation

All participants underwent amyloid PET using 18F-florbetapir. In this study, an amyloid-PET imaging system (Biograph Horizon; Siemens Medical Solutions USA, Inc., Malvern, PA) was used. Reconstructions were performed using time-of-flight (TOF) measurements; pixel sizes in the axial plane and the slice thickness were 1.03 and 2.0 mm, respectively. Iterative reconstruction employed a three-dimensional ordered subset expectation maximization (OSEM) algorithm with eight iterations and 10 subsets. Attenuation correction was used, and post-filtering was applied using the Gaussian filter at 4 mm.

PET images were visually assessed by two licensed physicians, and visual interpretation was prioritized as the primary determinant of amyloid positivity. Visual reads were performed independently, and any discrepancies were resolved by consensus. According to published visual-read criteria for florbetapir PET, a scan was considered visually positive if it showed (a) two or more cortical regions (each larger than a single gyrus) in which the grey-white matter boundary was indistinct or lost, or (b) any region where grey-matter (GM) uptake clearly exceeded adjacent white matter [14].

Quantitative CL values were calculated according to the CL project standardization procedure [8]. Standardized uptake value ratio (SUVR) was computed as the mean uptake in a cortical composite volume-of-interest (VOI) divided by the mean uptake in a reference region (whole cerebellum). Tracer-specific SUVRs were then linearly transformed to a Pittsburgh compound-B (PiB)-equivalent SUVR using published calibration equations and finally converted to CL by anchoring 0 CL to the mean of young controls (YC) and 100 CL to the mean of typical AD patients:

$$CL = 100 \times \frac{(SUVR_{PiB\text{-}equivalent} - \mu_{YC})}{(\mu_{AD} - \mu_{YC})}$$

In this expression, the Greek letter “μ” (mu) indicates the mean cortical SUVR for each reference population. CL values can be negative (below the YC mean) or exceed 100 (above the AD mean). In the phase 3 donanemab trial (TRAILBLAZER-ALZ 2), screening amyloid positivity required >37 CL, and dose-stopping for amyloid clearance was triggered at <11 CL (single scan) or 11-<25 CL (two consecutive scans) [15]. Company materials also note that <24.1 CL aligns with a negative visual read. Participants were classified as PET-positive when the visual read met the criteria, even when CL values were between 0 and 24.1.

MRI acquisition and VSRAD analysis

All participants underwent three-dimensional T1 (longitudinal relaxation time)-weighted MRI scans using a 1.5-Tesla Philips Achieva scanner (3D T1 first field echo, repetition time = 9.4 ms, echo time = 4.0 ms, voxel size = 0.94 × 1.17 × 2.00 mm³) within one month of amyloid PET imaging.

Quantitative assessment of medial temporal atrophy was performed using VSRAD version 2.1, employing the default normalization and smoothing parameters (8-mm Gaussian kernel) based on the 1.5-T normal database [4-7]. Four indices were extracted automatically from the standardized database: (1) severity, defined as the mean Z-score within a predefined hippocampal VOI encompassing the bilateral hippocampi and adjacent entorhinal cortices, reflecting the degree of medial temporal GM loss; (2) extent of GM (%), the percentage of total cerebral GM voxels exceeding a Z-score threshold of 2, reflecting the overall burden of cortical atrophy; (3) extent of VOI (%), the percentage of voxels within the VOI exceeding the same threshold, indicating the proportion of the medial temporal region of interest (ROI) affected; and (4) VOI/GM ratio, the ratio of the extent of VOI (%) to the extent of GM (%), providing a relative measure of focal (medial temporal) versus global cortical atrophy. Higher severity and extent values indicate greater atrophy, whereas the VOI/GM ratio reflects the relative distribution of focal versus diffuse cortical atrophy [4-7].

Statistical analysis

Given the small sample size, findings were interpreted as preliminary and exploratory. Analyses were performed using Python 3.11. Continuous variables were summarized as mean ± standard deviation (SD). Group differences between PET-positive and PET-negative participants were examined using Welch’s t-test. Correlations between CL values and each VSRAD index were tested with Spearman’s rank correlation. Receiver operating characteristic (ROC) curves were generated, and area under the curve (AUC) values were calculated. Two-tailed p values < 0.05 were considered statistically significant.

## Results

Baseline demographic, cognitive, and PET characteristics of the amyloid PET-positive (n = 8) and PET-negative (n = 8) groups are summarized in Table 1. No significant differences were found in age, sex ratio, MMSE, or Clinical Dementia Rating (CDR)-Global Score between the two groups. As expected, both CL and SUVR values were markedly higher in the PET-positive group (p < 0.001). These findings confirm that the groups were well matched in demographic and cognitive variables, differing only in amyloid burden.

**Table 1:** Baseline demographic, cognitive, and amyloid-PET characteristics of PET-positive and PET-negative groups. Values represent mean ± SD (range) or counts. Statistical analysis was performed using Welch’s t-test for continuous variables and Fisher’s exact test for categorical variables. A two-tailed p-value < 0.05 was considered statistically significant.

Variable	PET-positive (n = 8)	PET-negative (n = 8)	p value
Age (years)	73.9 ± 2.9 (70–78)	74.4 ± 7.9 (63–87)	0.88
Sex (male/female)	3/5	2/6	> 0.99
MMSE	28.4 ± 0.7 (28–30)	28.6 ± 0.5 (28–29)	0.60
CDR-Global Score	0.5 ± 0.0	0.5 ± 0.0	–
Centiloid	9.7 ± 5.9	−5.8 ± 6.2	< 0.001
Whole-Brain SUVR	1.10 ± 0.04	1.01 ± 0.03	< 0.001

MRI-derived morphometric indices were analyzed using VSRAD. Although group comparisons did not reach statistical significance, mean values of VSRAD severity (1.40 ± 0.60 vs 0.80 ± 0.60; p = 0.066) and the VOI/GM ratio (6.58 ± 5.84 vs 2.12 ± 3.69; p = 0.093) were higher in the PET-positive group, suggesting a trend toward greater medial temporal atrophy (Table 2). Extent-related indices (extent of GM and extent of VOI) showed no significant group differences.

**Table 2:** Comparison of VSRAD indices between the amyloid PET-positive and PET-negative groups. Values are presented as mean ± standard deviation (SD). Group comparisons were performed using Welch’s t-test. A two-tailed p-value < 0.05 was considered statistically significant.

VSRAD Index	Positive Mean ± SD	Negative Mean ± SD	t	p value
Severity	1.40 ± 0.60	0.80 ± 0.60	1.99	0.066
Extent of GM (%)	3.22 ± 1.44	3.70 ± 1.18	-0.73	0.477
Extent of VOI (%)	24.41 ± 23.67	8.48 ± 15.21	1.60	0.135
VOI/GM Ratio	6.58 ± 5.84	2.12 ± 3.69	1.83	0.093

Correlation analysis revealed significant positive associations between CL values and several VSRAD indices, including severity (r = 0.55, p = 0.028), extent of VOI (%) (r = 0.63, p = 0.008), and VOI/GM ratio (r = 0.68, p = 0.003). These findings suggest that higher amyloid burden is modestly linked to greater regional atrophy within the hippocampal VOI.

**Figure 1:**
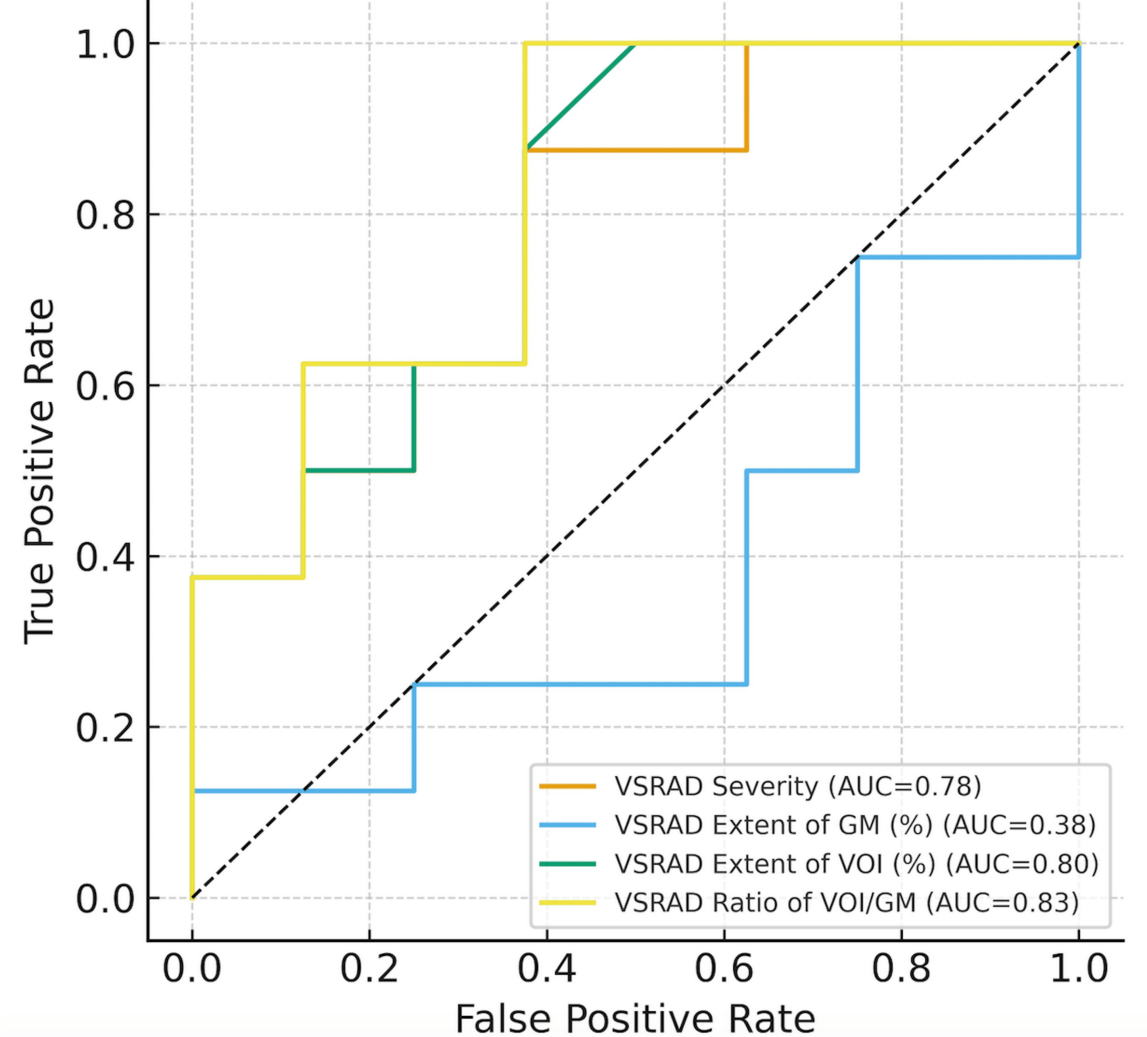
Receiver-operating-characteristic (ROC) curves showing the discriminative ability of VSRAD indices for predicting amyloid PET positivity. The VOI/GM ratio achieved the highest AUC (0.83), followed by the extent of VOI (0.80) and severity (0.78). The diagonal line indicates the reference (AUC = 0.5).

ROC curve analyses demonstrated fair discriminative ability of VSRAD indices for predicting PET positivity, with AUC values of 0.78 for severity, 0.80 for extent of VOI (%), and 0.83 for the VOI/GM ratio. Among them, VOI/GM showed the highest predictive performance (Figure 1), indicating its potential utility as an MRI-derived biomarker reflecting subclinical amyloid pathology.

## Discussion

This preliminary study examined whether MRI-derived medial temporal morphometry can anticipate amyloid PET positivity in individuals with preserved cognition (MMSE 28-30). Our main findings were as follows: (i) VSRAD severity and the VOI/GM ratio showed a non-significant trend toward higher values in the PET-positive group; (ii) CL values correlated positively with medial-temporal-focused indices (severity, extent of VOI, VOI/GM); and (iii) among VSRAD measures, the VOI/GM ratio achieved the best discrimination (AUC 0.83). Our findings suggest a possible association between VSRAD-based medial temporal atrophy and amyloid PET positivity in cognitively normal individuals. Given the small sample size and cross-sectional design, these results should be interpreted as preliminary and hypothesis-generating. Larger prospective studies are warranted to validate these associations and clarify the potential of MRI-derived morphometric markers for preclinical AD screening.

At the dementia stage of AD, hippocampal atrophy is a well-established diagnostic marker and tracks disease severity and progression [1-3,16]. Standardized tools, such as VSRAD, operationalize patient-level detection of medial temporal atrophy in clinical practice [4-7]. However, evidence is less consistent in cognitively normal or “near-healthy” cohorts. Multiple community-based studies have reported modest associations between cortical amyloid burden and either smaller hippocampal volumes or faster medial temporal atrophy, yet effect sizes vary and sometimes attenuate after adjustment for age, apolipoprotein E ε4 status, or concomitant neurodegeneration markers [9-12]. Conversely, voxel-based approaches focusing solely on medial temporal regions have been reported to show limited capability to detect Aβ pathology per se, underscoring methodological sensitivity and the biological subtlety of preclinical change [13].

Our preliminary results align with this mixed literature: global atrophy indices (extent of GM) did not differentiate PET groups, whereas region-focused indices linked to the hippocampal VOI did - both in group trends and in their correlation with CL. Mechanistically, this pattern is compatible with the amyloid/tau/ neurodegeneration (A/T/N) framework in which amyloid (A⁺) alone is not strongly neurodegenerative; hippocampal atrophy is thought to be more tightly coupled to tau-mediated (T⁺) processes and downstream neurodegeneration (N⁺). The positive - yet only moderate - associations we observed likely reflect early or heterogeneous coupling between amyloid deposition and medial temporal structural change, potentially modulated by incipient tau spread within entorhinal-hippocampal circuits and by network-level vulnerability of default mode network hubs.

From a practical standpoint, medial-temporal-weighted indices (e.g., VOI/GM ratio) may serve as a low-cost triage tool to enrich for amyloid positivity when PET availability is limited. Such MRI signals should not replace amyloid biomarkers but could help prioritize candidates for further testing or for trial screening in preclinical AD. Methodologically, our findings argue for region-specific morphometry rather than global volumetrics when probing early disease, careful control of reference databases, and harmonized reporting of thresholds and effect sizes alongside CL.

This study had several limitations, including the small sample size (n = 16), cross-sectional design, and lack of tau or other biomarker data. The MMSE scores of participants were within the normal range, which may have introduced a ceiling effect in cognitive measures. Despite these constraints, the exploratory analysis provides an initial step toward linking structural MRI findings to amyloid pathology in cognitively normal individuals. The ceiling effect of MMSE and the relatively short interval between MRI and PET may have obscured potential longitudinal associations. Moreover, tau PET and plasma or cerebrospinal fluid biomarkers were not evaluated, which could help clarify whether the observed MRI-Aβ relationships are direct or mediated by tau pathology. Future studies with larger, longitudinal cohorts incorporating A/T/N biomarkers and cognitive follow-up are warranted to determine whether VSRAD-derived indices - particularly the VOI/GM ratio - can predict progression to symptomatic stages or the rate of cognitive decline among amyloid-positive but cognitively normal individuals.

## Conclusions

While hippocampal morphometry is validated in dementia-stage AD, our data suggest that, even in near-healthy cohorts, medial temporal indices derived from routine MRI show measurable alignment with amyloid burden. Leveraging these indices alongside CL-based PET may improve early risk stratification in preclinical AD. Given the small sample size and cross-sectional design, these results should be interpreted as preliminary and hypothesis-generating. Larger prospective studies are warranted to validate these associations and clarify the potential of MRI-derived morphometric markers for early-stage or preclinical AD screening.
